# Supplementary material for: Prediction of continuous amyloid positron emission tomography with fluid measures of phosphorylated tau and β-amyloid
Source: EMBO Mol Med. 2025 Dec 1;18(1):217–31. doi: 10.1038/s44321-025-00348-7 (PMC12808103; doi:10.1038/s44321-025-00348-7)
Supplement: Supplementary file 1 — Table EV1 [file 44321_2025_348_MOESM1_ESM.docx]

|  | Level | BF2 | BF2-P-MS | BF2-P-IA | BF2-C-IA | BF2-C-MS | BF2-Initial |
| --- | --- | --- | --- | --- | --- | --- | --- |
| n |  | 1140 | 1053 | 907 | 980 | 254 | 499 |
| Age [mean (sd)] |  | 66.5 (13.3) | 66.3 (13.5) | 64.86 (13.44) | 65.07 (13.28) | 71.11 (8.98) | 65.9  (13.7) |
| Sex [n (%)] | Male | 549 (48.1) | 510  (48.4) | 451 (49.72) | 487 (49.69) | 133 (52.36) | 244  (48.9) |
|  | Female | 591 (51.8) | 543  (51.6) | 456 (50.28) | 493 (50.31) | 121 (47.64) | 255  (51.1) |
| Education [mean (sd)] |  | 12.9 (3.69) | 12.9 (3.70) | 12.93 (3.64) | 12.88 (3.63) | 12.23 (3.75) | 12.8 (3.64) |
| Cognitive status [n (%)] | CN | 579 (50.8) | 548  (52.0) | 430 (47.41) | 447 (45.61) | 123 (48.43) | 263  (52.7) |
|  | SCD | 217 (19.0) | 195  (18.5) | 185 (20.40) | 212 (21.63) | 34  (13.39) | 93  (18.6) |
|  | MCI | 325 (28.5) | 292  (27.7) | 276 (30.43) | 305 (31.12) | 88  (34.65) | 141  (28.3) |
|  | dementia | 19  (1.70) | 18  (1.71) | 16  (1.76) | 16  (1.63) | 9  (3.54) | 2  (0.4) |
| *APOE* ε4 alleles [n (%)] | 0 | 542 (47.5) | 516  (49.0) | 465 (51.27) | 499  (50.92) | 104 (40.94) | 256  (49.3) |
|  | 1 | 517 (45.4) | 461  (43.8) | 371 (40.90) | 408 (41.63) | 127 (50.00) | 214  (42.9) |
|  | 2 | 81 (7.1) | 76  (7.21) | 71  (7.83) | 73  (7.45) | 23  (9.06) | 29  (5.81) |
| MMSE [mean (sd)] |  | 28.3 (1.89) | 28.3 (1.91) | 28.28 (1.93) | 28.28 (1.92) | 27.94 (2.16) | 28.3 (1.81) |
| ADAS-cog [mean (sd)] |  | 3.74 (2.73) | 3.69  (2.74) | 3.72 (2.81) | 3.74 (2.79) | 4.52 (2.86) | 3.76  (2.78) |
| Aβ PET, SUVR [mean (sd)] |  | 1.10 (0.303) | 1.10  (0.299) | 1.10 (0.30) | 1.10 (0.30) | 1.24 (0.32) | 1.09  (0.30) |
| Aβ PET, status [n (%)] | Normal | 717 (62.9) | 674  (64.0) | 588 (64.83) | 628 (64.08) | 84  (33.07) | 337  (67.5) |
|  | Abnormal | 423 (37.1) | 379  (36.0) | 319 (35.17) | 352 (35.92) | 170 (66.93) | 162  (32.5) |
| Plasma %P-tau217 [mean (sd)] |  |  | 1.32  (1.21) | 0.25 (0.23) |  |  | 1.34  (1.30) |
| CSF P-tau217 [mean (sd)] |  |  |  |  | 13.18 (17.64) | 5.63 (3.41) | 13.8 (19.4) |
| CSF Aβ42/Aβ40 [mean (sd)] |  | 0.0903 (0.0319) | 0.0907  (0.0318) | 0.09 (0.03) | 0.09 (0.03) | 0.07 (0.03) | 0.0920  (0.0321) |

***Table EV1.*** *Complete demographics of participants from the Swedish BioFINDER-2 Study. Initial model development was done on a subset with as complete data as possible, BF2-Initial. For further analyses, four distinct sub-cohorts were defined and used, differentiated by the availability of P-tau217 measurements in plasma (P-MS: plasma samples analyzed by MS-method and P-IA: plasma samples analyzed by Lilly immunoassay) and CSF (C-IA: CSF samples analyzed by Lilly immunoassay and C-MS: CSF samples analyzed by MS-method). Note that P-tau217 is quantified in varying units across these sources, with WashU using percent mean and Lilly using picograms per milliliter (pg/ml).*
