# Supplementary material for: Prediction of continuous amyloid positron emission tomography with fluid measures of phosphorylated tau and β-amyloid
Source: EMBO Mol Med. 2025 Dec 1;18(1):217–31. doi: 10.1038/s44321-025-00348-7 (PMC12808103; doi:10.1038/s44321-025-00348-7)
Supplement: Supplementary file 2 — Table EV2 [file 44321_2025_348_MOESM2_ESM.docx]

| **Feature Combination** | **1** | **2** | **3** | **4** | **5** | **6** | **7** | **8** | **9** | **10** | **11** |
| --- | --- | --- | --- | --- | --- | --- | --- | --- | --- | --- | --- |
| **Plasma %P-tau217** | **x** |  |  |  | **x** |  | **x** |  | **x** | **x** | **x** |
| **CSF Aβ42/Aβ40** | **x** | **x** | **x** | **x** |  | **x** |  |  |  | **x** | **x** |
| **Age** | **x** | **x** | **x** | **x** | **x** | **x** | **x** | **x** | **x** | **x** | **x** |
| **Plasma P-tau217** |  | **x** |  |  |  |  |  |  |  |  |  |
| **Plasma P-tau217 Lilly** |  |  | **x** |  |  |  |  |  |  |  |  |
| **CSF P-tau217** |  |  |  | **x** |  |  |  |  |  | **x** | **x** |
| **Plasma Aβ42/Aβ40** |  |  |  |  |  |  | **x** | **x** | **x** | **x** | **x** |
| **Plasma P-tau231** |  |  |  |  |  |  |  |  | **x** | **x** | **x** |
| **Plasma %P-tau205** |  |  |  |  |  |  |  |  | **x** | **x** | **x** |
| **Plasma %P-tau181** |  |  |  |  |  |  |  |  | **x** | **x** | **x** |
| **ADAS** |  |  |  |  |  |  |  |  |  |  | **x** |
| **Cog status** |  |  |  |  |  |  |  |  |  |  | **x** |
| **R^2^** | 0.826 | 0.812 | 0.807 | 0.791 | 0.786 | 0.704 | 0.782 | 0.299 | 0.784 | 0.833 | 0.841 |
| **MAPE** | 6.4% | 6.7% | 6.9% | 6.9% | 7.1% | 8.2% | 7.2% | 14.8% | 7.3% | 6.3% | 6.3% |
| **MAPE (Aβ-)** | 4.7% | 4.7% | 5.1% | 5.0% | 5.3% | 5.9% | 5.5% | 12.8% | 5.8% | 4.7% | 4.7% |
| **MAPE (Aβ+)** | 9.8% | 10.8% | 10.6% | 11.0% | 10.9% | 12.8% | 10.6% | 19.0% | 10.4% | 9.6% | 9.6% |

***Table EV2.*** *Control experiments of final feature selection. “x” means the feature was included in the combination. Note that the statistics here are from the cross-validated training sets and therefore differ slightly from test-set statistics reported in the main text.*
