# Supplementary material for: Prediction of continuous amyloid positron emission tomography with fluid measures of phosphorylated tau and β-amyloid
Source: EMBO Mol Med. 2025 Dec 1;18(1):217–31. doi: 10.1038/s44321-025-00348-7 (PMC12808103; doi:10.1038/s44321-025-00348-7)
Supplement: Supplementary file 3 — Appendix [file 44321_2025_348_MOESM3_ESM.pdf]

Appendix

Appendix	Page
Appendix Table S1. *Complete feature names in the model development and feature engineering.*	*2*
Appendix Table S2. *Description of model configurations in initial model selection.*	*3*
Appendix Table S3. *Performance of various machine learning models trained on BF2-Initial in initial model selection.*	*4*
Appendix Table S4. *Control experiments of final feature selection.*	*5*
**Appendix Figures**	
Appendix Fig S1. *Detailed analysis of feature importance in the initial model selection evaluated by three different methods.*	*6*
Appendix Fig S2. *A comparison to the Figure S1 when no CSF data was included in the initial model selection.*	*7*
Appendix Fig S3. *A comparison to the Figure S1 when no CSF data and plasma P-tau217 was included in the initial model selection.*	*8*
Appendix Fig S4. *The fluctuation of feature importance when analyzing two different types of regressors trained on BF2-P-MS.*	*9*
Appendix Fig S5. *A two-step procedure for clearer analyses of feature importance derived from the Extra Trees Regressor with 4 features*	*10*
Appendix Fig S6. *Validation on BF1 for the Extra Trees Regressor trained on Aβ-positive samples in the two-step procedure.*	*11*
Appendix Fig S7. *Performance of the best predictive model (Gradient Boosting Regressor) trained and tested on BF2-P-MS.*	*12*
Appendix Fig S8. *A 2-step procedure for clearer analyses of feature importance derived from the Gradient Boosting Regressor with 2 features*	*13*
Appendix Fig S9. *The probability of a predicted value located in different observation groups.*	*14*

Appendix Table S1. *Complete feature names in the model development and feature engineering.*

**Feature List**
CSF Aβ42/Aβ40 Roche
Plasma %P-tau181 WashU
Plasma %P-tau205 WashU
Plasma %P-tau217 WashU
Plasma P-tau181 WashU
Plasma P-tau205 WashU
Plasma P-tau217 WashU
Plasma P-tau217 Lilly
Plasma Aβ42/Aβ40 WashU
CSF P-tau181 Roche
CSF P-tau217 Lilly
Plasma GFAP UGOT
Plasma NfL UGOT
Plasma NTA UGOT
Plasma P-tau231 UGOT
Age
APOE
Education
Sex
ADAS
Animal fluency
Cognitive status
Diagnosis status
PACC
MMSE
Symbol digit
Trail-Making

Appendix Table S2. *Description of model configurations in initial model selection.*

Model Structure	Hyper-parameters
**ExtraTreesRegressor**	max_depth=11
**GradientBoostingRegressor**	max_depth=2, n_estimators=50
**XGBRegressor**	max_depth=2, n_estimators=50, eta=0.09
**BaggingRegressor**	n_estimators=100
**RandomForestRegressor**	max_depth=5
**AdaBoostRegressor**	n_estimators=450
**DecisionTreeRegressor**	max_depth=3
**Ridge**	alpha=0.5
**SVR**	kernel='poly'
**KNeighborsRegressor**	n_neighbors=10

Appendix Table S3. *Performance of various machine learning models trained on BF2-Initial in initial model selection. All the models listed were tuned properly and cross-validated in 5 folds.*

Model	Train R2	Validation R2
**ExtraTreesRegressor**	0.970	0.827
**XGBRegressor**	0.923	0.812
**GradientBoostingRegressor**	0.938	0.808
**RandomForestRegressor**	0.949	0.805
**BaggingRegressor**	0.974	0.804
**DecisionTreeRegressor**	0.880	0.776
**AdaBoostRegressor**	0.932	0.774
**Ridge**	0.764	0.695
**KNeighborsRegressor**	0.584	0.485
**SVR**	0.649	0.462

Appendix Table S4. *Model selection for the regression within the Aβ-positive range. Every regressor was properly tuned and cross-validated in 5 folds.*

Model	Train R2	Validation R2	Train MAPE	Validation MAPE
**ExtraTreesRegressor**	0.638	0.362	0.081	0.110
**GradientBoostingRegressor**	0.617	0.352	0.085	0.111
**XGBRegressor**	0.590	0.352	0.088	0.112
**RandomForestRegressor**	0.730	0.343	0.071	0.110
**BaggingRegressor**	0.903	0.332	0.042	0.110
**Ridge**	0.348	0.302	0.116	0.119
**AdaBoostRegressor**	0.568	0.296	0.104	0.123
**SVR**	0.334	0.288	0.114	0.118
**DecisionTreeRegressor**	0.503	0.248	0.096	0.119
**KNeighborsRegressor**	0.388	0.244	0.113	0.125

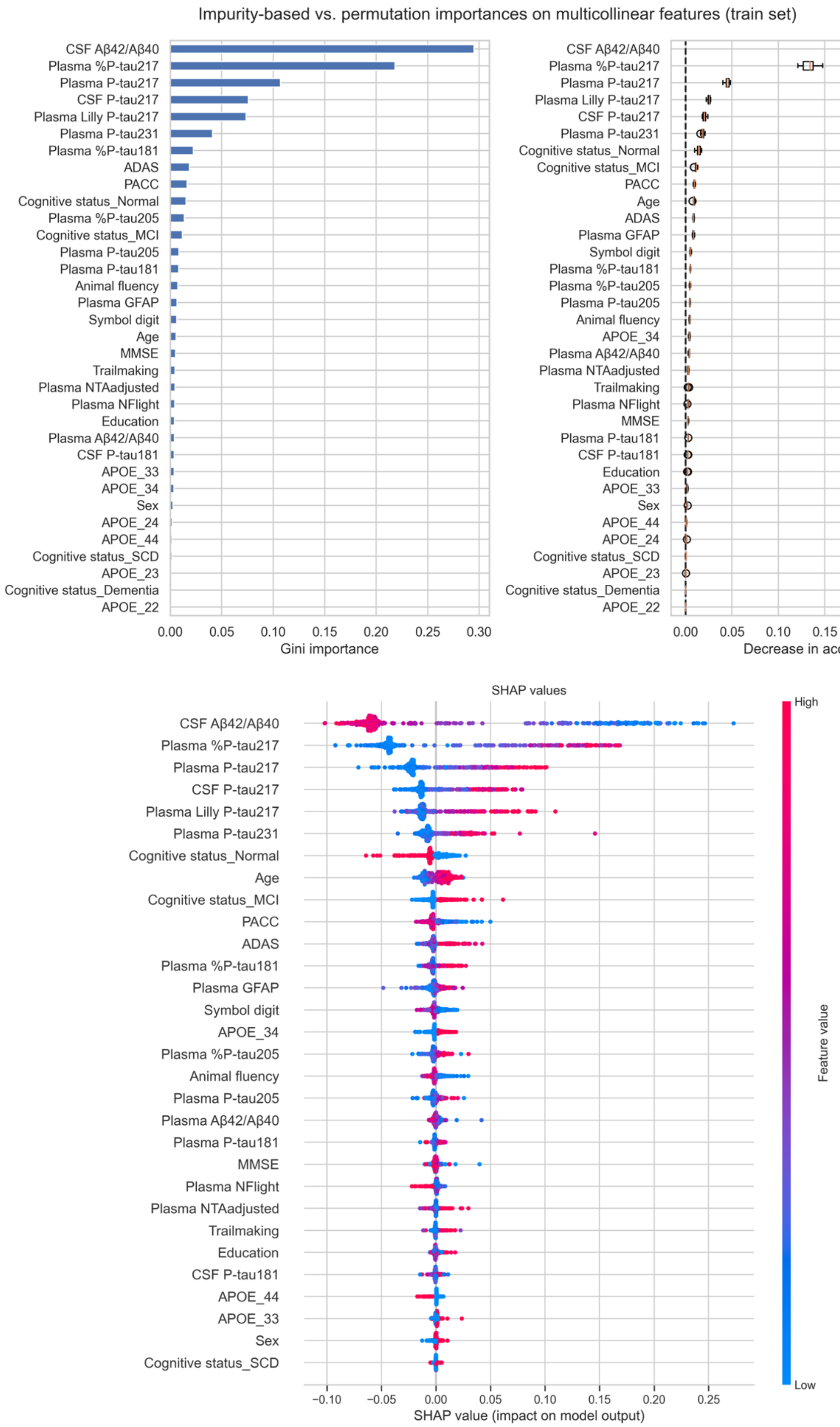

Appendix Figure S1. *Detailed analysis of feature importance in the initial model selection evaluated by three different methods.*

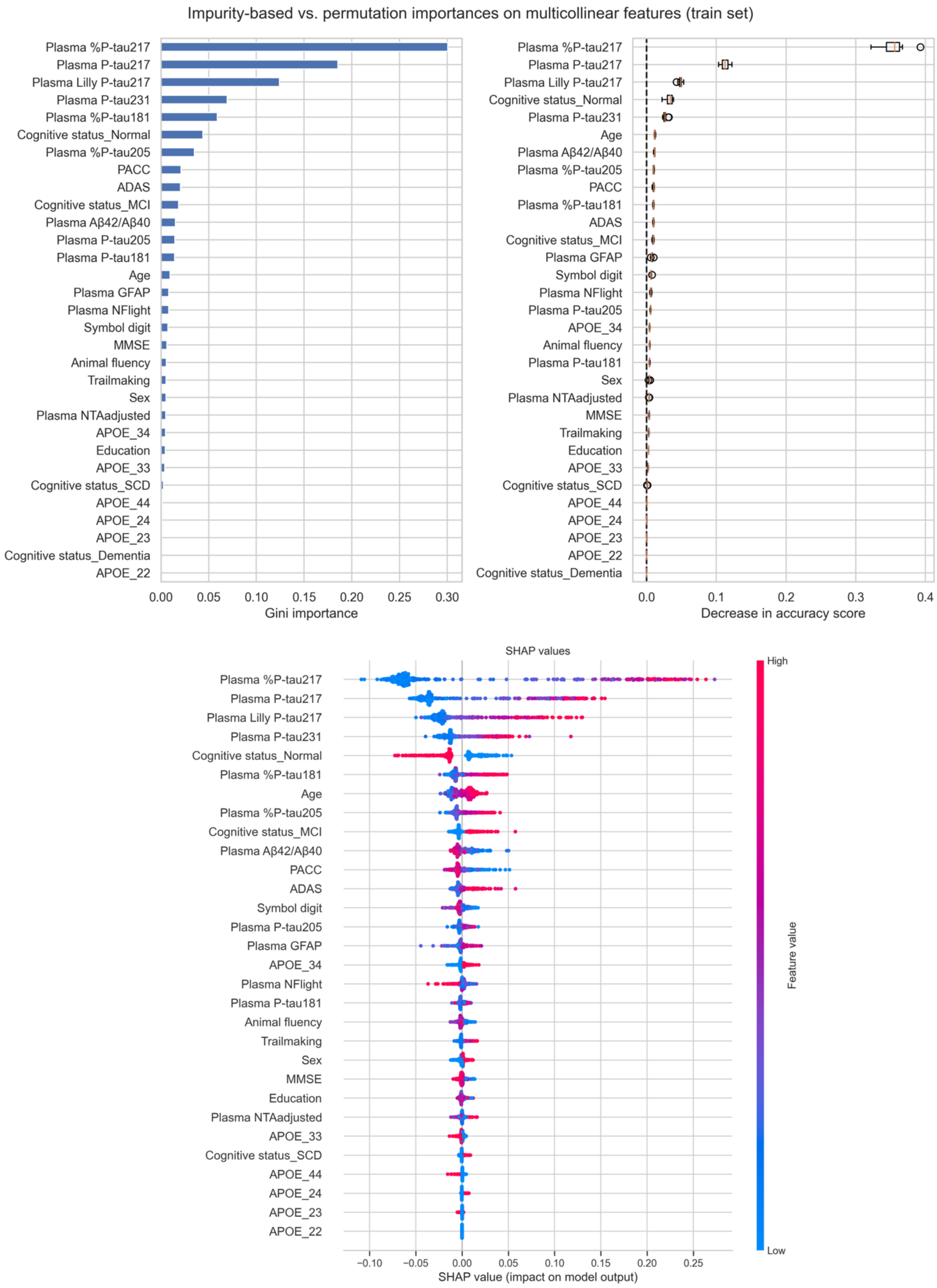

Appendix Figure S2. *A comparison to the Appendix Figure S1 when no CSF data was included in the initial model selection.*

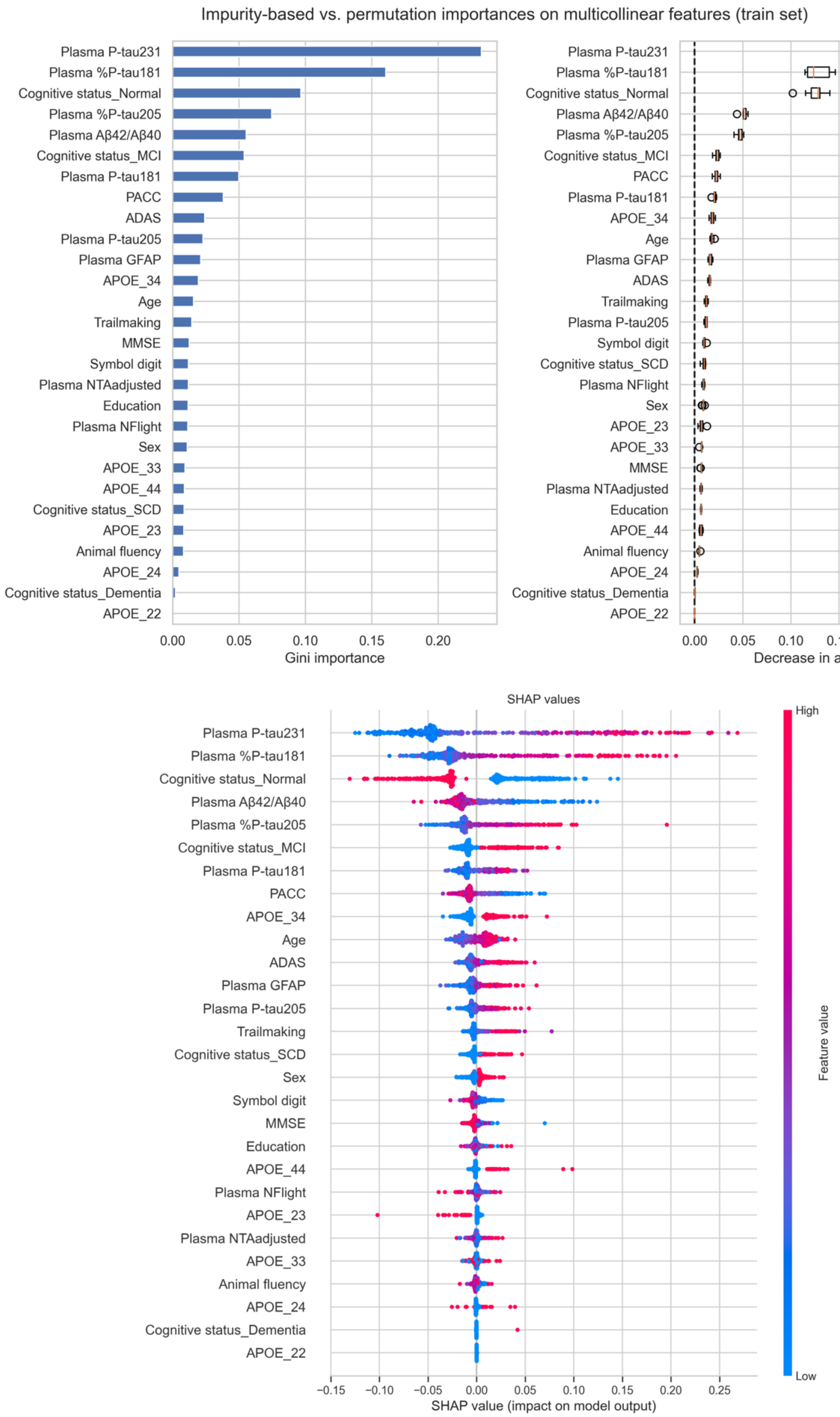

Appendix Figure S3. *A comparison to the Appendix Figure S1 when no CSF data and plasma P-tau217 was included in the initial model selection.*

A

B

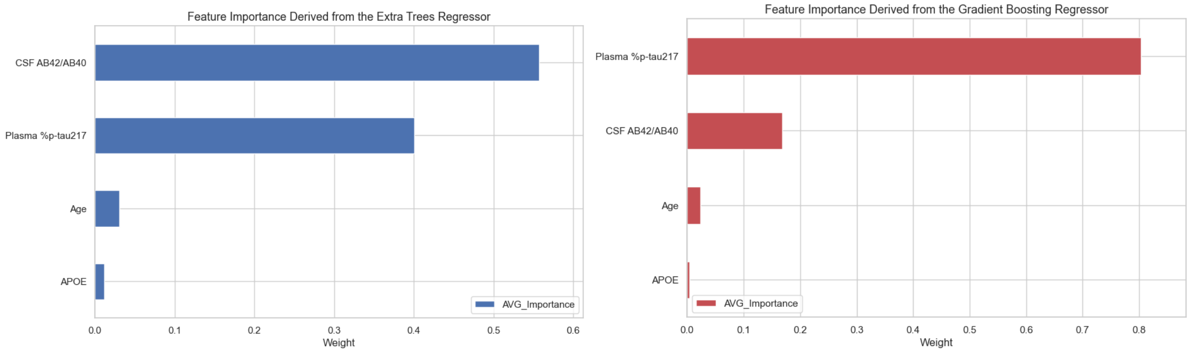

Appendix Figure S4. *The fluctuation of feature importance when analyzing two different types of regressors trained on BF2-P-MS. The Extra Trees Regressor in panel A presents CSF Aβ42/Aβ40 as the dominant predictor, while the Gradient Boosting Regressor in panel B presents plasma %P-tau217 as the most influential predictor.*

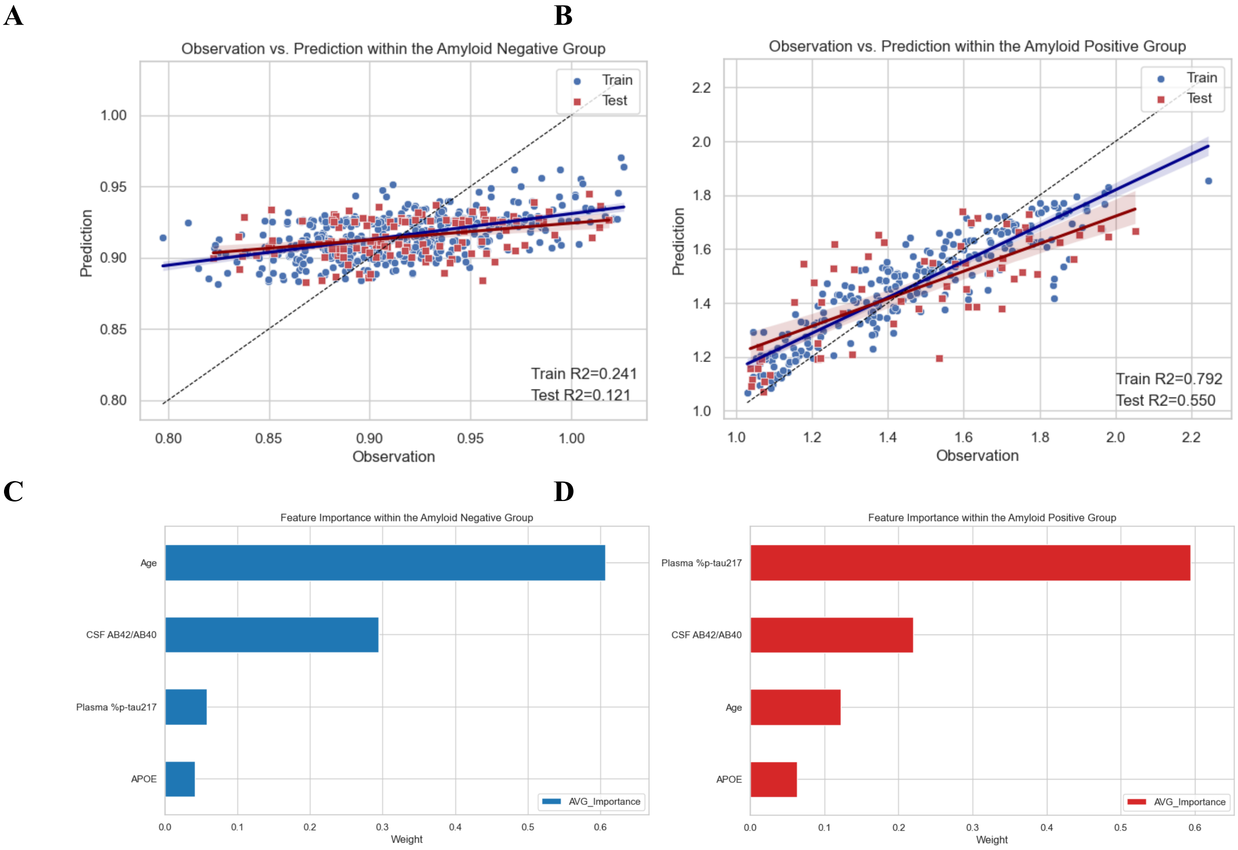

Appendix Figure S5. *A two-step procedure for clearer analyses of feature importance derived from the Extra Trees Regressor with four features ('CSF Aβ42/Aβ40', 'plasma %P-tau217', 'age' and 'APOE genotype'). A-B) Observation vs. prediction within the Aβ-negative and positive group respectively. C) Receiver operating characteristic Curve for the binary classifier. D) The feature importance for both the classifier and the positive regressor.*

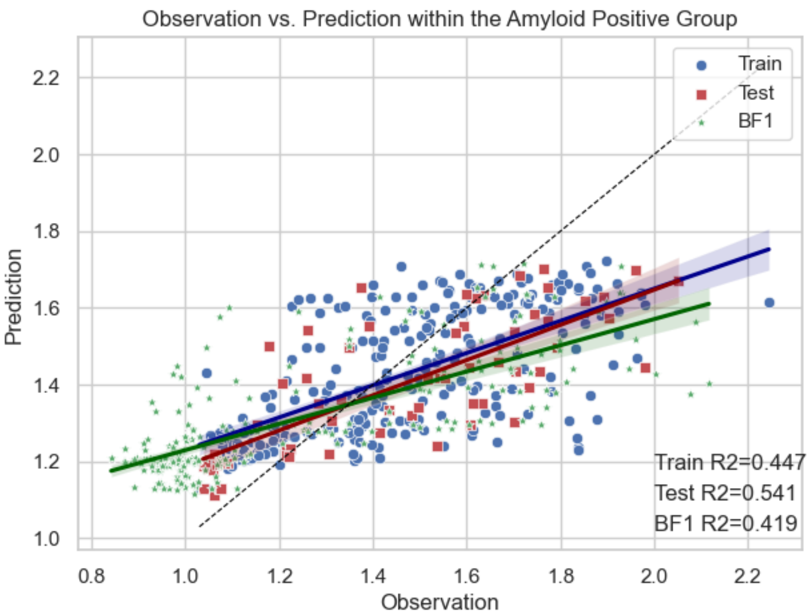

Appendix Figure S6. *Validation on BF1 for the Extra Trees Regressor trained on Aβ-positive samples in the two-step procedure.*

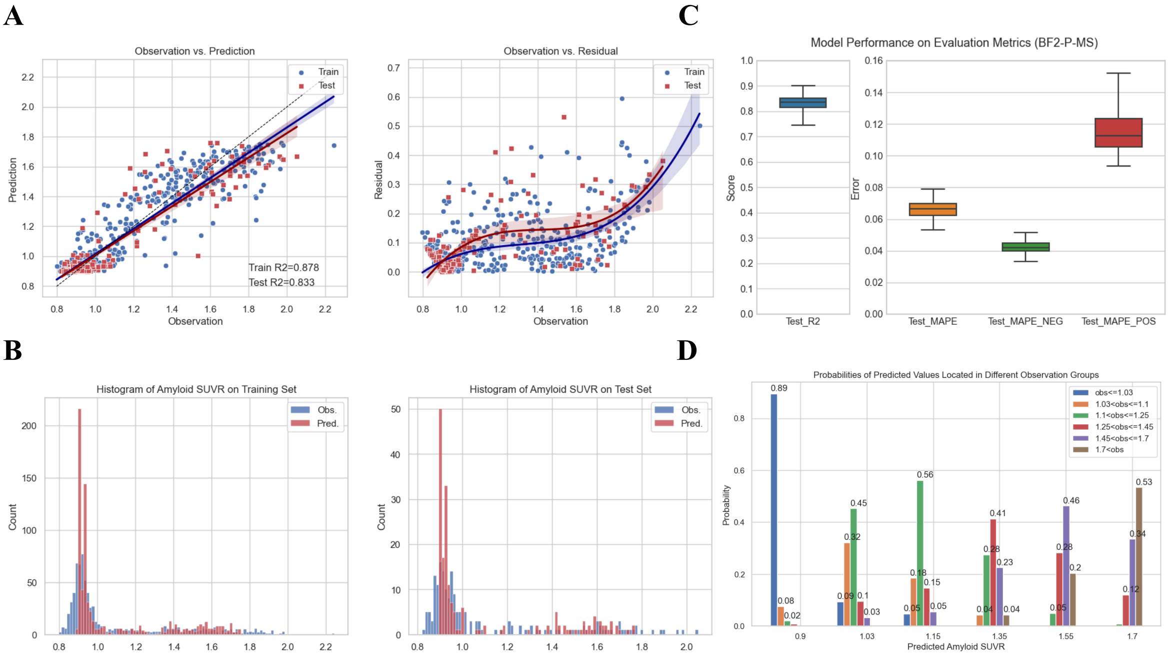

Appendix Figure S7. *Performance of the best predictive model (Gradient Boosting Regressor) trained and tested on BF2-P-MS. A) Observations versus predictions and residuals. B) Distributions of observed and predicted Aβ-PET SUVR on training and test set respectively. C) Results on the test set with* R^2 *and MAPE on the whole Aβ-PET range, and MAPE_NEG and MAPE_POS on the negative and positive ranges, respectively. The results were generated using a bootstrapping method where we resampled 80 samples with replacement for 100 iterations from the test set in order to evaluate the variance of the test set. D) Probabilities of the model's predictions (6 specific cutting points) falling within a number of observation groups.*

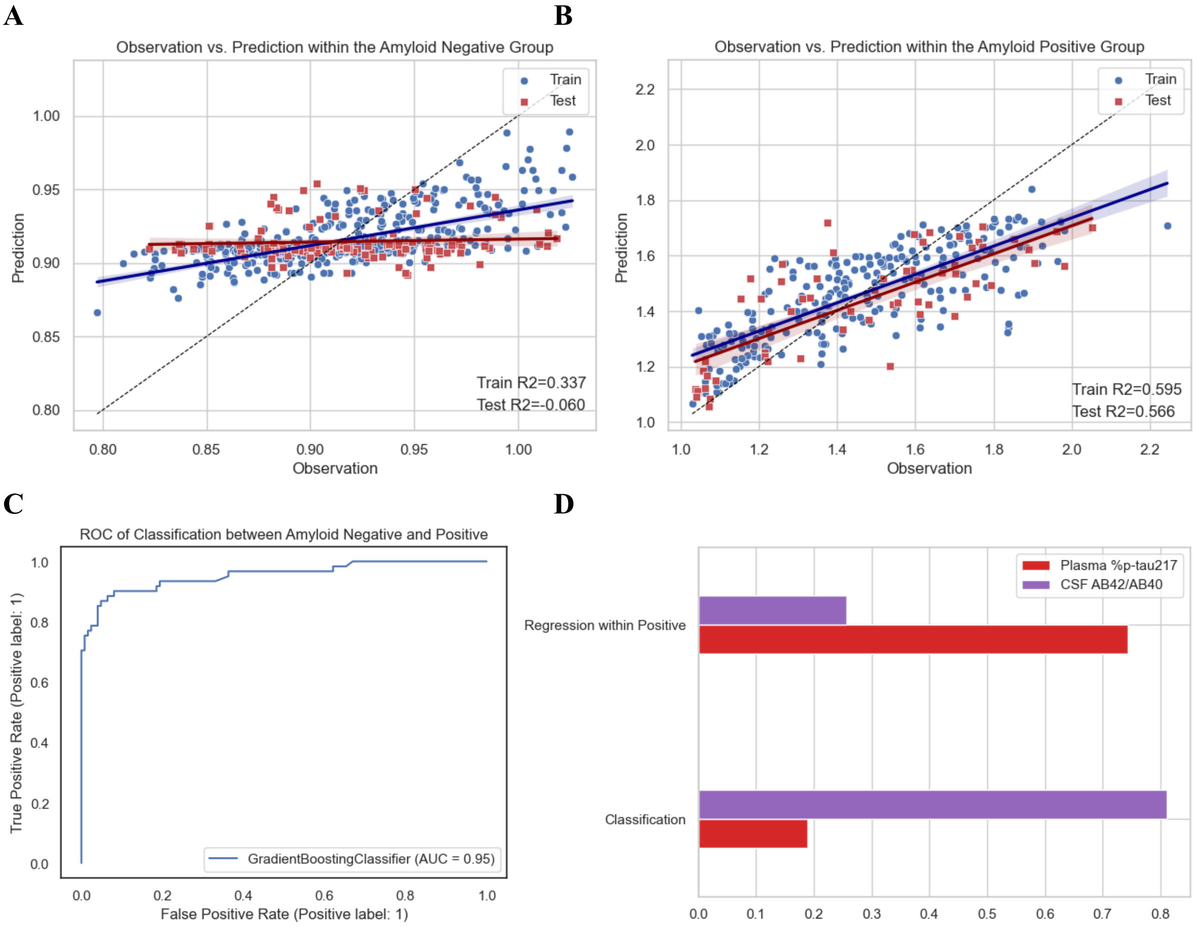

Appendix Figure S8. *A two-step procedure for clearer analyses of feature importance derived from the Gradient Boosting Regressor with two features ('CSF Aβ42/Aβ40' and 'plasma %P-tau217'). A-B) Observation vs. prediction within the Aβ-negative and positive group respectively. C) Receiver operating characteristic Curve for the binary classifier. D) The feature importance for both the classifier and the positive regressor.*

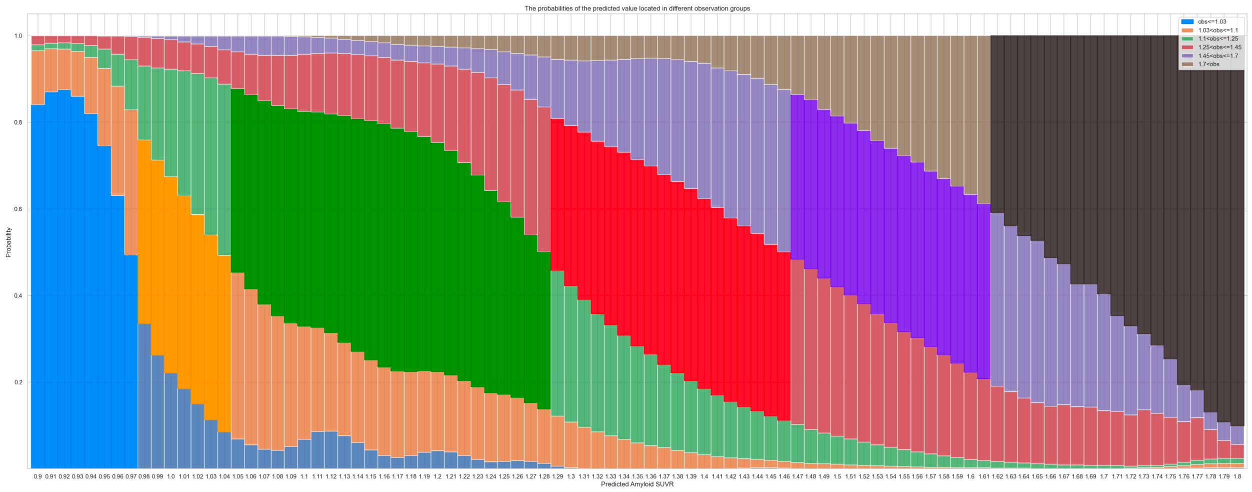

Appendix Figure S9. *The probability of a predicted value located in different observation groups. Each color represents a specific observation group. The highlighted area signifies the observation group with the highest probability at each specific predicted amyloid value. In short, the observation is most likely to be, e.g., lower or equal to 1.03 (y<=1.03) when the prediction is lower or equal to 0.97 (ŷ<=0.97); 1.03<y<=1.1 when 0.97<ŷ<=1.04; 1.1<y<1.25 when 1.04< ŷ<=1.28; 1.25<y<=1.45 when 1.28< ŷ<=1.46; 1.45<y<=1.7 when 1.46< ŷ<=1.61; 1.7<y when 1.61< ŷ<=1.8.*
