# Supplementary material for: Prediction of continuous amyloid positron emission tomography with fluid measures of phosphorylated tau and β-amyloid
Source: EMBO Mol Med. 2025 Dec 1;18(1):217–31. doi: 10.1038/s44321-025-00348-7 (PMC12808103; doi:10.1038/s44321-025-00348-7)
Supplement: Supplementary file 10 — Expanded View Figures [file 44321_2025_348_MOESM10_ESM.pdf]

# Expanded View Figures

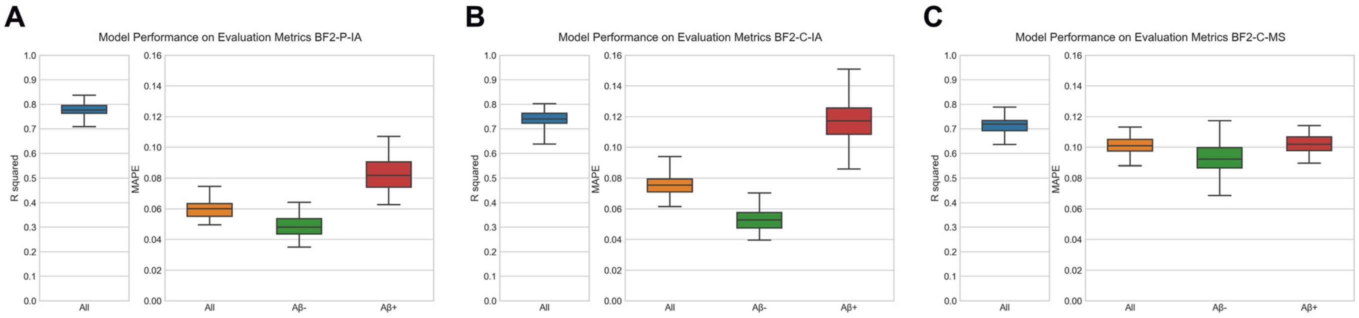


**Figure EV1. Performance of models trained on sub-cohorts.**

Panels (**A–C**) illustrate the bootstrapping evaluation results on the test sets for the sub-cohorts BF2-P-IA (**A**), BF2-C-IA (**B**), and BF2-C-MS (**C**), with $R^2$ and MAPE on the whole Aβ-PET range, and the metrics MAPE_POS and MAPE_NEG on the Aβ-positive and Aβ-negative range, respectively. The box plots show the quartiles of these 100 iterations (IQR = Q3–Q1), with median as center line, and whiskers extending to minimum and maximum points.
